# 5-(4-Pentyl­phen­yl)-1,3,4-thia­diazol-2-amine

**DOI:** 10.1107/S1600536809022594

**Published:** 2009-06-17

**Authors:** Yao Wang, Rong Wan, Feng Han, Peng Wang

**Affiliations:** a Department of Applied Chemistry, College of Science, Nanjing University of Technology, No. 5 Xinmofan Road, Nanjing, Nanjing 210009, People’s Republic of China

## Abstract

The title compound, C_13_H_17_N_3_S, was synthesized by the reaction of 4-pentyl­benzoic acid and thio­semicarbazide. The dihedral angle between the thia­diazole and phenyl rings is 29.9 (2)°. An intra­molecular C—H⋯S inter­action is observed. In the crystal, inter­molecular N—H⋯N hydrogen bonding links the mol­ecules into centrosymmetric dimers.

## Related literature

For general background to the biological activity of 1,3,4-thia­diazole derivatives, see: Nakagawa *et al.* (1996 ▶); Wang *et al.* (1999 ▶). For bond-length data, see: Allen *et al.* (1987 ▶).
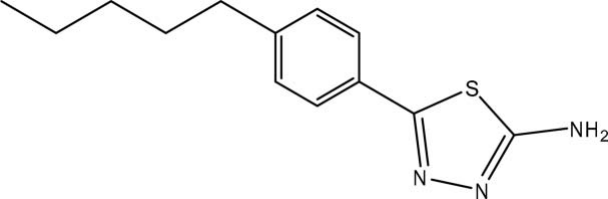


## Experimental

### 

#### Crystal data

C_13_H_17_N_3_S
*M* *_r_* = 247.36
Monoclinic, 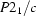

*a* = 14.012 (3) Å
*b* = 9.1300 (18) Å
*c* = 10.938 (2) Å
β = 100.64 (3)°
*V* = 1375.2 (5) Å^3^
*Z* = 4
Mo *K*α radiation
μ = 0.22 mm^−1^
*T* = 293 K
0.20 × 0.10 × 0.05 mm

#### Data collection

Enraf–Nonius CAD-4 diffractometer
Absorption correction: ψ scan (North *et al.*, 1968 ▶) *T*_min_ = 0.958, *T*_max_ = 0.989
2596 measured reflections
2492 independent reflections
1405 reflections with *I* > 2σ(*I*)
*R*_int_ = 0.023
3 standard reflections every 200 reflections intensity decay: 1%

#### Refinement

*R*[*F* ^2^ > 2σ(*F* ^2^)] = 0.066
*wR*(*F* ^2^) = 0.179
*S* = 1.00
2492 reflections
148 parameters
1 restraint
H-atom parameters constrained
Δρ_max_ = 0.18 e Å^−3^
Δρ_min_ = −0.52 e Å^−3^

### 

Data collection: *CAD-4 EXPRESS* (Enraf–Nonius, 1989 ▶); cell refinement: *CAD-4 EXPRESS*; data reduction: *XCAD4* (Harms & Wocadlo, 1995 ▶); program(s) used to solve structure: *SHELXS97* (Sheldrick, 2008 ▶); program(s) used to refine structure: *SHELXL97* (Sheldrick, 2008 ▶); molecular graphics: *SHELXTL* (Sheldrick, 2008 ▶); software used to prepare material for publication: *SHELXL97*.

## Supplementary Material

Crystal structure: contains datablocks global, I. DOI: 10.1107/S1600536809022594/hg2519sup1.cif

Structure factors: contains datablocks I. DOI: 10.1107/S1600536809022594/hg2519Isup2.hkl

Additional supplementary materials:  crystallographic information; 3D view; checkCIF report

## Figures and Tables

**Table 1:** Hydrogen-bond geometry (Å, °)

*D*—H⋯*A*	*D*—H	H⋯*A*	*D*⋯*A*	*D*—H⋯*A*
C8—H8*A*⋯S	0.93	2.81	3.177 (4)	104
N3—H3*A*⋯N2^i^	0.86	2.15	3.006 (4)	173

Symmetry code: (i) 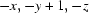.

**Fig. 1.**
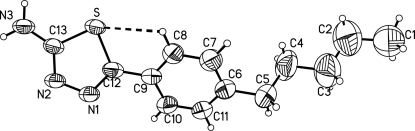
A view of the molecular structure of (I). Displacement ellipsoids are drawn at the 50% probability level. Dashed lines indicate C—H···S short contact distance.

**Fig. 2.**
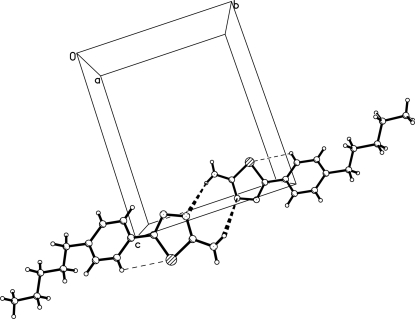
Partial packing view showing the hydrogen-bonded network. Dashed lines indicate intermolecular N—H···N hydrogen bond.

## supplementary crystallographic information

### Comment

1,3,4-Thiadiazole derivatives represent an interesting class of compounds possessing broad spectrum biological activities (Nakagawa *et al.*, 1996). These compounds are known to exhibit diverse biological effects, such as insecticidal, fungicidal activities (Wang *et al.*, 1999). The structure of the title compound, (I), is shown in Fig. 1, in which the bond lengths (Allen *et al.*, 1987) and angles are generally within normal ranges. The dihedral angle between the thiadiazole and phenyl ring is 29.90 (19)°. An intramolecular C—H···S interaction is observed (Fig. 1). There is intermolecular N—H···N hydrogen bond (Fig. 2), forming chains along the *c* axis. The intermolecular N—H···N hydrogen bond creates centrosymmetric dimers.

### Experimental

4-Pentylbenzoic acid (5 mmol) and thiosemicarbazide (5 mmol) were mixed in a 25 ml flask, and kept in the oil bath at 90°C for 6 h. After cooling, the crude product (I) precipitated and was filtered. Pure compound (I) was obtained by crystallization from ethanol(20 ml). Crystals of (I) suitable for X-ray diffraction were obtained by slow evaporation of an acetone solution.

### Refinement

All H atoms bonded to the C atoms were placed geometrically at the distances of 0.93–0.97 Å and included in the refinement in riding motion approximation with *U*_iso_(H) = 1.2 or 1.5*U*_eq_ of the carrier atom.

### Crystal data

**Table:**

C_13_H_17_N_3_S	*F*(000) = 528
*M**_r_* = 247.36	*D*_x_ = 1.195 Mg m^−^^3^
Monoclinic, *P*2_1_/*c*	Melting point: 563 K
Hall symbol: -P 2ybc	Mo *K*α radiation, λ = 0.71073 Å
*a* = 14.012 (3) Å	Cell parameters from 25 reflections
*b* = 9.1300 (18) Å	θ = 8–12°
*c* = 10.938 (2) Å	µ = 0.22 mm^−^^1^
β = 100.64 (3)°	*T* = 293 K
*V* = 1375.2 (5) Å^3^	Block, colorless
*Z* = 4	0.20 × 0.10 × 0.05 mm

### Data collection

**Table:**

Enraf–Nonius CAD-4 diffractometer	1405 reflections with *I* > 2σ(*I*)
Radiation source: fine-focus sealed tube	*R*_int_ = 0.023
graphite	θ_max_ = 25.3°, θ_min_ = 1.5°
ω/2θ scans	*h* = −16→0
Absorption correction: ψ scan (North *et al.*, 1968)	*k* = 0→10
*T*_min_ = 0.958, *T*_max_ = 0.989	*l* = −12→13
2596 measured reflections	3 standard reflections every 200 reflections
2492 independent reflections	intensity decay: 1%

### Refinement

**Table:**

Refinement on *F*^2^	Primary atom site location: structure-invariant direct methods
Least-squares matrix: full	Secondary atom site location: difference Fourier map
*R*[*F*^2^ > 2σ(*F*^2^)] = 0.066	Hydrogen site location: inferred from neighbouring sites
*wR*(*F*^2^) = 0.179	H-atom parameters constrained
*S* = 1.00	*w* = 1/[σ^2^(*F*_o_^2^) + (0.09*P*)^2^] where *P* = (*F*_o_^2^ + 2*F*_c_^2^)/3
2492 reflections	(Δ/σ)_max_ < 0.001
148 parameters	Δρ_max_ = 0.18 e Å^−^^3^
1 restraint	Δρ_min_ = −0.52 e Å^−^^3^

### Special details

**Table:**

Geometry. All e.s.d.'s (except the e.s.d. in the dihedral angle between two l.s. planes) are estimated using the full covariance matrix. The cell e.s.d.'s are taken into account individually in the estimation of e.s.d.'s in distances, angles and torsion angles; correlations between e.s.d.'s in cell parameters are only used when they are defined by crystal symmetry. An approximate (isotropic) treatment of cell e.s.d.'s is used for estimating e.s.d.'s involving l.s. planes.
Refinement. Refinement of *F*^2^ against ALL reflections. The weighted *R*-factor *wR* and goodness of fit *S* are based on *F*^2^, conventional *R*-factors *R* are based on *F*, with *F* set to zero for negative *F*^2^. The threshold expression of *F*^2^ > σ(*F*^2^) is used only for calculating *R*-factors(gt) *etc*. and is not relevant to the choice of reflections for refinement. *R*-factors based on *F*^2^ are statistically about twice as large as those based on *F*, and *R*- factors based on ALL data will be even larger.

### Fractional atomic coordinates and isotropic or equivalent isotropic displacement parameters (Å^2^)

**Table:**

	*x*	*y*	*z*	*U*_iso_*/*U*_eq_	
S	0.10971 (8)	0.15085 (11)	0.16795 (8)	0.0627 (4)	
N1	0.0965 (2)	0.1921 (3)	−0.0647 (2)	0.0595 (9)	
N2	0.0606 (2)	0.3197 (3)	−0.0202 (2)	0.0596 (9)	
N3	0.0292 (3)	0.4194 (3)	0.1651 (2)	0.0721 (10)	
H3A	0.0054	0.4985	0.1290	0.087*	
H3B	0.0321	0.4085	0.2438	0.087*	
C1	0.4434 (5)	−0.9308 (7)	0.1391 (6)	0.143	
H1B	0.4664	−0.9713	0.2201	0.214*	
H1C	0.4927	−0.9409	0.0892	0.214*	
H1D	0.3860	−0.9820	0.1001	0.214*	
C2	0.4207 (5)	−0.7748 (8)	0.1509 (7)	0.165 (3)	
H2B	0.4800	−0.7243	0.1875	0.198*	
H2C	0.3761	−0.7660	0.2085	0.198*	
C3	0.3778 (5)	−0.6986 (7)	0.0342 (7)	0.142 (2)	
H3C	0.3235	−0.7561	−0.0083	0.170*	
H3D	0.4259	−0.6949	−0.0191	0.170*	
C4	0.3422 (5)	−0.5426 (6)	0.0510 (5)	0.135 (2)	
H4A	0.3003	−0.5452	0.1123	0.162*	
H4B	0.3981	−0.4828	0.0851	0.162*	
C5	0.2901 (4)	−0.4706 (5)	−0.0597 (4)	0.0891 (14)	
H5A	0.2378	−0.5344	−0.0983	0.107*	
H5B	0.3340	−0.4591	−0.1180	0.107*	
C6	0.2473 (3)	−0.3212 (4)	−0.0391 (4)	0.0714 (12)	
C7	0.1937 (3)	−0.3008 (4)	0.0533 (4)	0.0773 (13)	
H7A	0.1831	−0.3795	0.1031	0.093*	
C8	0.1557 (3)	−0.1657 (4)	0.0731 (4)	0.0683 (11)	
H8A	0.1218	−0.1544	0.1380	0.082*	
C9	0.1665 (3)	−0.0480 (4)	0.0002 (3)	0.0555 (9)	
C10	0.2190 (3)	−0.0673 (4)	−0.0951 (3)	0.0708 (12)	
H10A	0.2275	0.0106	−0.1467	0.085*	
C11	0.2583 (3)	−0.2025 (5)	−0.1123 (4)	0.0742 (12)	
H11A	0.2935	−0.2138	−0.1759	0.089*	
C12	0.1252 (3)	0.0956 (4)	0.0207 (3)	0.0548 (9)	
C13	0.0617 (3)	0.3139 (4)	0.0991 (3)	0.0552 (9)	

### Atomic displacement parameters (Å^2^)

**Table:**

	*U*^11^	*U*^22^	*U*^33^	*U*^12^	*U*^13^	*U*^23^
S	0.0965 (8)	0.0583 (6)	0.0329 (5)	0.0097 (6)	0.0112 (4)	0.0063 (4)
N1	0.084 (2)	0.059 (2)	0.0356 (14)	0.0052 (17)	0.0115 (14)	0.0018 (14)
N2	0.087 (2)	0.061 (2)	0.0311 (14)	0.0078 (17)	0.0113 (14)	0.0040 (13)
N3	0.125 (3)	0.060 (2)	0.0326 (15)	0.023 (2)	0.0179 (17)	0.0032 (14)
C1	0.143	0.143	0.143	0.000	0.024	0.000
C2	0.162 (7)	0.139 (6)	0.190 (8)	0.050 (5)	0.024 (6)	0.014 (6)
C3	0.138 (6)	0.117 (5)	0.170 (7)	0.033 (4)	0.024 (5)	0.002 (5)
C4	0.157 (6)	0.103 (4)	0.129 (5)	0.064 (4)	−0.016 (4)	−0.027 (4)
C5	0.112 (4)	0.073 (3)	0.080 (3)	0.014 (3)	0.013 (3)	−0.015 (3)
C6	0.089 (3)	0.066 (3)	0.055 (2)	0.008 (2)	0.001 (2)	−0.009 (2)
C7	0.110 (4)	0.059 (3)	0.067 (3)	−0.003 (2)	0.025 (3)	0.002 (2)
C8	0.094 (3)	0.055 (2)	0.059 (2)	0.005 (2)	0.024 (2)	0.0064 (19)
C9	0.065 (2)	0.063 (2)	0.0365 (17)	0.002 (2)	0.0058 (16)	−0.0017 (17)
C10	0.098 (3)	0.068 (3)	0.050 (2)	0.007 (2)	0.024 (2)	0.003 (2)
C11	0.095 (3)	0.078 (3)	0.051 (2)	0.015 (3)	0.017 (2)	−0.004 (2)
C12	0.067 (2)	0.059 (2)	0.0358 (17)	−0.0042 (19)	0.0041 (16)	0.0006 (16)
C13	0.075 (3)	0.054 (2)	0.0349 (17)	0.0020 (19)	0.0045 (16)	0.0047 (16)

### Geometric parameters (Å, °)

**Table:**

S—C12	1.739 (3)	C4—C5	1.451 (6)
S—C13	1.746 (3)	C4—H4A	0.9700
N1—C12	1.293 (4)	C4—H4B	0.9700
N1—N2	1.392 (4)	C5—C6	1.524 (5)
N2—C13	1.304 (4)	C5—H5A	0.9700
N3—C13	1.333 (4)	C5—H5B	0.9700
N3—H3A	0.8600	C6—C11	1.373 (5)
N3—H3B	0.8600	C6—C7	1.378 (6)
C1—C2	1.471 (6)	C7—C8	1.377 (5)
C1—H1B	0.9600	C7—H7A	0.9300
C1—H1C	0.9600	C8—C9	1.362 (5)
C1—H1D	0.9600	C8—H8A	0.9300
C2—C3	1.480 (8)	C9—C10	1.393 (5)
C2—H2B	0.9700	C9—C12	1.467 (5)
C2—H2C	0.9700	C10—C11	1.379 (5)
C3—C4	1.531 (7)	C10—H10A	0.9300
C3—H3C	0.9700	C11—H11A	0.9300
C3—H3D	0.9700		
			
C12—S—C13	87.26 (17)	C4—C5—C6	115.6 (4)
C12—N1—N2	113.7 (3)	C4—C5—H5A	108.4
C13—N2—N1	112.3 (3)	C6—C5—H5A	108.4
C13—N3—H3A	120.0	C4—C5—H5B	108.4
C13—N3—H3B	120.0	C6—C5—H5B	108.4
H3A—N3—H3B	120.0	H5A—C5—H5B	107.4
C2—C1—H1B	109.5	C11—C6—C7	117.2 (4)
C2—C1—H1C	109.5	C11—C6—C5	122.0 (4)
H1B—C1—H1C	109.5	C7—C6—C5	120.8 (4)
C2—C1—H1D	109.5	C8—C7—C6	121.0 (4)
H1B—C1—H1D	109.5	C8—C7—H7A	119.5
H1C—C1—H1D	109.5	C6—C7—H7A	119.5
C1—C2—C3	116.1 (6)	C9—C8—C7	121.8 (4)
C1—C2—H2B	108.3	C9—C8—H8A	119.1
C3—C2—H2B	108.3	C7—C8—H8A	119.1
C1—C2—H2C	108.3	C8—C9—C10	118.0 (4)
C3—C2—H2C	108.3	C8—C9—C12	121.7 (3)
H2B—C2—H2C	107.4	C10—C9—C12	120.3 (3)
C2—C3—C4	115.0 (6)	C11—C10—C9	119.7 (4)
C2—C3—H3C	108.5	C11—C10—H10A	120.2
C4—C3—H3C	108.5	C9—C10—H10A	120.2
C2—C3—H3D	108.5	C6—C11—C10	122.4 (4)
C4—C3—H3D	108.5	C6—C11—H11A	118.8
H3C—C3—H3D	107.5	C10—C11—H11A	118.8
C5—C4—C3	116.5 (5)	N1—C12—C9	125.3 (3)
C5—C4—H4A	108.2	N1—C12—S	113.3 (3)
C3—C4—H4A	108.2	C9—C12—S	121.4 (3)
C5—C4—H4B	108.2	N2—C13—N3	124.8 (3)
C3—C4—H4B	108.2	N2—C13—S	113.4 (3)
H4A—C4—H4B	107.3	N3—C13—S	121.7 (2)
			
C12—N1—N2—C13	−1.4 (5)	C5—C6—C11—C10	179.4 (4)
C1—C2—C3—C4	172.2 (6)	C9—C10—C11—C6	0.4 (7)
C2—C3—C4—C5	−173.4 (6)	N2—N1—C12—C9	−179.7 (3)
C3—C4—C5—C6	174.6 (5)	N2—N1—C12—S	0.8 (4)
C4—C5—C6—C11	133.0 (5)	C8—C9—C12—N1	−150.2 (4)
C4—C5—C6—C7	−48.4 (7)	C10—C9—C12—N1	30.3 (6)
C11—C6—C7—C8	−2.0 (7)	C8—C9—C12—S	29.2 (5)
C5—C6—C7—C8	179.4 (4)	C10—C9—C12—S	−150.3 (3)
C6—C7—C8—C9	2.2 (7)	C13—S—C12—N1	−0.1 (3)
C7—C8—C9—C10	−1.0 (6)	C13—S—C12—C9	−179.6 (3)
C7—C8—C9—C12	179.5 (4)	N1—N2—C13—N3	−178.2 (4)
C8—C9—C10—C11	−0.2 (6)	N1—N2—C13—S	1.3 (4)
C12—C9—C10—C11	179.3 (4)	C12—S—C13—N2	−0.7 (3)
C7—C6—C11—C10	0.7 (7)	C12—S—C13—N3	178.8 (3)

### Hydrogen-bond geometry (Å, °)

**Table:**

*D*—H···*A*	*D*—H	H···*A*	*D*···*A*	*D*—H···*A*
C8—H8A···S	0.93	2.81	3.177 (4)	104
N3—H3A···N2^i^	0.86	2.15	3.006 (4)	173

Symmetry codes: (i) −*x*, −*y*+1, −*z*.
